# Systems infection biology: a compartmentalized immune network of pig spleen challenged with *Haemophilus parasuis*

**DOI:** 10.1186/1471-2164-14-46

**Published:** 2013-01-22

**Authors:** Ming Zhao, Xiang-dong Liu, Xin-yun Li, Hong-bo Chen, Hui Jin, Rui Zhou, Meng-jin Zhu, Shu-hong Zhao

**Affiliations:** 1 Key Lab of Agricultural Animal Genetics, Breeding, and Reproduction of Ministry of Education, Huazhong Agricultural University, Wuhan, 430070, PR China; 2 School of Animal Science and Nutritional Engineering, Wuhan Polytechnic University, Wuhan, Hubei, 430023, PR China; 3 Division of Animal Infectious Disease, State Key Laboratory of Agricultural Microbiology, Huazhong Agricultural University, Wuhan, 430070, PR China

**Keywords:** Pig model, *Haemophilus parasuis*, Spleen, Immunogenome, Network, Quantitative topology, Scale-free, C1R

## Abstract

**Background:**

Network biology (systems biology) approaches are useful tools for elucidating the host infection processes that often accompany complex immune networks. Although many studies have recently focused on *Haemophilus parasuis*, a model of Gram-negative bacterium, little attention has been paid to the host's immune response to infection. In this article, we use network biology to investigate infection with *Haemophilus parasuis* in an *in vivo* pig model.

**Results:**

By targeting the spleen immunogenome, we established an expression signature indicative of *H. parasuis* infection using a PCA/GSEA combined method. We reconstructed the immune network and estimated the network topology parameters that characterize the immunogene expressions in response to *H. parasuis* infection. The results showed that the immune network of *H. parasuis* infection is compartmentalized (not globally linked). Statistical analysis revealed that the reconstructed network is scale-free but not small-world. Based on the quantitative topological prioritization, we inferred that the C1R-centered clique might play a vital role in responding to *H. parasuis* infection.

**Conclusions:**

Here, we provide the first report of reconstruction of the immune network in *H. parasuis*-infected porcine spleen. The distinguishing feature of our work is the focus on utilizing the immunogenome for a network biology-oriented analysis. Our findings complement and extend the frontiers of knowledge of host infection biology for *H. parasuis* and also provide a new clue for systems infection biology of Gram-negative *bacilli* in mammals.

## Background

Glässer's disease is caused by *Haemophilus parasuis* (shortened as *H. parasuis* or HPS), a model Gram-negative *bacillus*. This disease is an important cause of economic loss in the world's pig industry, which is clinically characterized by fibrinous polyserositis, polyarthritis and meningitis [1]. To date, the major focus of studies of porcine Glässer's disease has centered on clinical symptoms, pathology and diagnosis, susceptibility and epidemiology, pathogenic biology, vaccine development, and evaluation of virulence-associated factors [2-6]. Many of these investigations have highly focused on the major aspects of biology and pathogenesis for the *H. parasuis* bacterium itself. Aside from several recent studies [7-11], the molecular mechanisms of the pig host that are involved in the response to the H. parasuis invasion have not been well addressed. More importantly, the pig is an excellent biomedical model because it has a closer phylogenetic and physiological relationship to humans than rodent models [12]. In addition to being a potential asset for undiscovered clinical and therapeutic needs, pigs infected with *H. parasuis* could also serve as mammalian and human models for bacterial infectious diseases.

It is well known that the immune genes (hereafter referred to as immunogenes) have played central roles in the regulation of pathogen-induced host processes *in vivo*, including those of Glässer's disease. Systems biology (also referred to as network biology) approaches have brought a research paradigm for infectious diseases; for example, a systems biology program was recently initiated by the National Institute of Allergy and Infectious Diseases [13]. Systems biology investigations of the transcriptome of host immunogenome could provide a profound exploration of the molecular events occurring, for example, the three- or even four-dimensional relationships between genes during a response to pathogen infection. This would increase our understanding of host resistance/susceptibility genes, immune response mechanisms, and molecular basis of host-pathogen interactions [14,15]. So, the systems biology approaches can also provide us with powerful tools for uncovering the molecular immune mechanisms that defend against *H. parasuis* infection.

Custom-build gene chips have been widely applied in a variety of investigations [16-21]. On many occasions, a reduced fragment of microarray data could work more efficiently to reveal more subtle insights into the target biological phenomena than the non-reduced global genome data do [22-24]. As a consequence, an analysis focusing on immunogenes could give a more precise exploration of the transcriptomic landscape of infection-induced immune processes in hosts.

In the body's immune system, spleen is an important target organ for studies of immune mechanisms. It has been well documented that the spleen is a crucial immune organ to protect the body against a variety of diseases and infections [25-27]. The spleen, known as the blood cleaner for its role in capturing foreign antigens and destroying old red blood cells, is made up of a variety of immune cells and blood cells, including B cells, T cells, macrophages, dendritic cells, natural killer cells and red blood cells [28-31]. When migratory macrophages and dendritic cells bring antigens to the spleen, the immune cells (e.g., T- and B-lymphocytes) become activated and trigger a series of immune responses [32-35]. Although not obligatory for survival, it has been proven that the spleen plays a key role in mounting immune responses to antigens, and in the absence of the spleen, the body would be more susceptible to infections [36]. Consequently, the spleen is one of the ideal organ models for studying host immune responses to pathogenic challenges, including the *H. parasuis* infection.

Our previous study has used the Affymetrix Porcine Genechip™ to profile the differentially expressed genes between spleens with and without administrations with the *H. parasuis*[10]. There were totally 931 differentially expressed transcripts, of which only a small fragment has been annotated as immunogenes. The result showed that the unfocused global expression profiling based on a full-genome array couldn’t reveal the subtle roles of immunogenes. In the present study, we aim to clarify the subtle roles of immunogenes in the host response to *H. parasuis* challenge. Using the pig (*Sus scrofa*) as an *in vivo* model, we first characterized the microarray expression dataset of the spleen's immunogenome. Based on the partitioned immunogenome dataset, we performed a comprehensive immunomic analysis, which included reconstruction of the immune network and evaluation of network parameters and quantitative topological properties. Our investigation revealed a vital network component in response to *H. parasuis* infection. To our knowledge, this is the first network biology analysis of the spleen immunogenome upon challenge with a Gram-negative bacterium in mammals.

## Results and Discussion

### Characterization of the immunogene dataset

We used the GeneChip® Porcine Genome Array (Affymetrix) to measure gene expressions of porcine spleen from three normal and three *H. parasuis*-infected samples from six separate piglets. By extracting immune pathways from KEGG and reactome databases (see the Additional file 1), a total of 1,999 transcripts from the 20,201 transcripts arrayed on the chip were targeted as immunogenes according to the pathway annotation results. The basic annotation information of Affymetrix probesets and corresponding transcripts of immunogenes is shown in the Additional file 2. Among the subsection of 1,999 transcripts of immunogenes, a total of 1,115 transcripts were detected to call Present in both normal and *H. parasuis*-infected samples. Additional file 3: Table S1 gives the descriptive statistical parameters used to evaluate the expression of the signals of the immunogenes on at least one chip using the mas5calls method.

**Figure 1:**
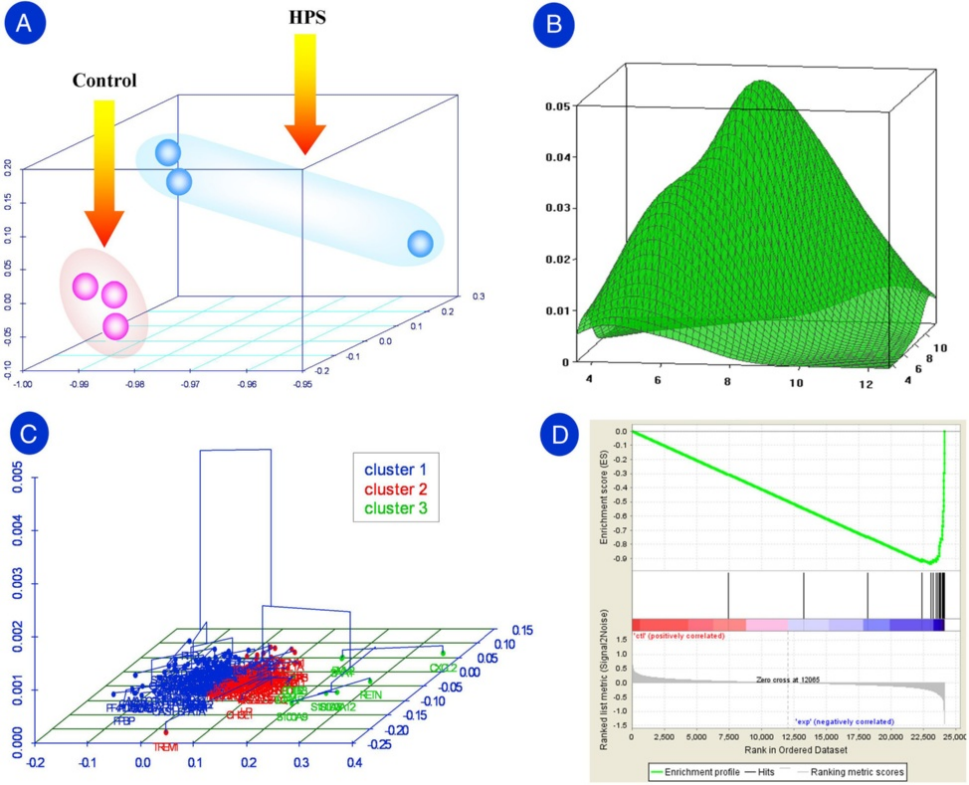
**Exploratory multivariate evaluation of the immunogene dataset. (A)** 3D plot of samples under the coordinates of the first three principal components. **(B)** Evaluation of joint densities: an example for the 3D plot of joint density between samples 2 and 6. **(C)** Hierarchical clustering on the factor map for immunogenes: 3D dendrogram of the first principal component. Here, the optimal level of division calculated suggests three clusters. **(D)** Enrichment plot for the gene set with the largest positive loadings on the second principal component. Profile of the running ES score and positions of GeneSet members on the rank-ordered list.

The dataset of immunogenes partitioned from the Affymetrix Porcine Genome Genechip was evaluated by exploratory multivariate analysis. First, the principal components analysis (PCA) revealed that a total of 6 principal components were detected, and their standard deviations were estimated to be 2.279, 0.710, 0.427, 0.248, 0.183 and 0.155, respectively. With the cumulative proportion up to 0.950, the major variation of dataset could be explained by the first two principal components. A 3D plot for the 6 chips (samples) under the coordinates of the first three principal components is given in Figure 1A. The between-chip joint densities of immunogenes were also evaluated, and Figure 1B gave an example for a 3D plot of the joint density between the samples 2 and 6. The symmetry of 3D plot of the between-chip joint densities can be used to reveal the technical stableness of microarray experiments. Here, all of the 3D plots had a symmetrical appearance, which means that our microarray experiments were technically reliable. Based on the Euclidean distance, Figure 1C also provides the 3D tree map for the hierarchical clustering of immunogenes under the first principal component [37].

**Figure 2:**
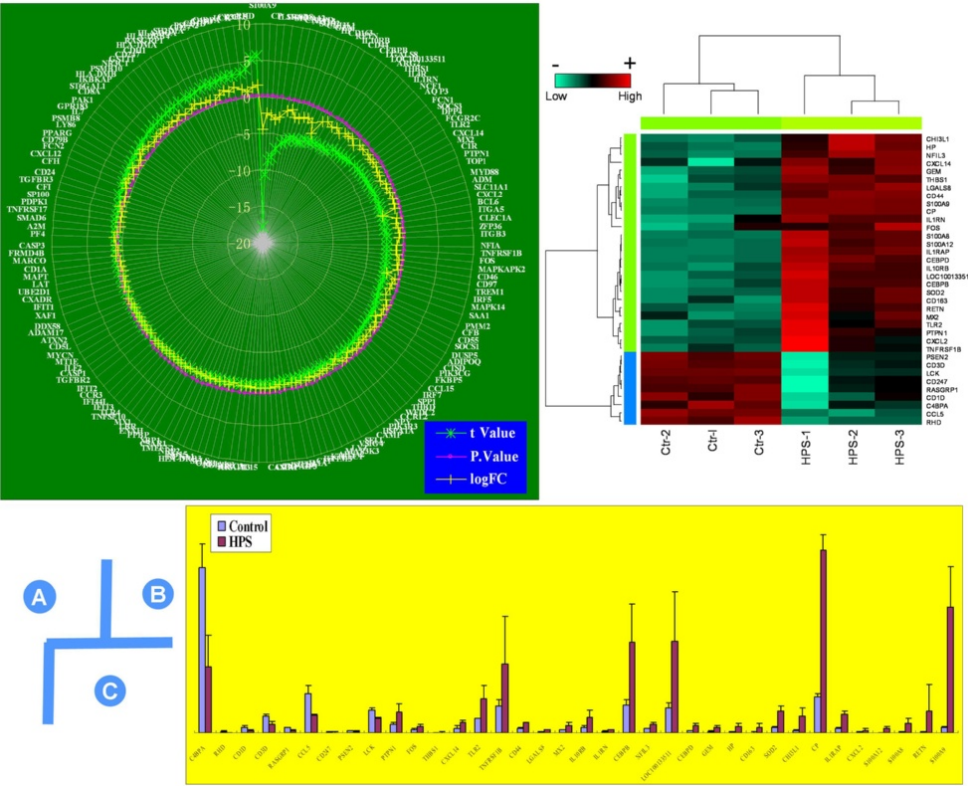
**Evaluation plots for differentially expressed immunogenes. (A)** Radial plots of t values, p values and logFC values for the differential expression test. The blue, red and green lines indicate t values, logFC values and p values, respectively. **(B)** Two-way hierarchical clustering of differentially expressed immunogenes. Rows represent genes and columns represent samples. Ctr-1, Ctr-2 and Ctr-3 were the control samples, and HPS-1, HPS-2 and HPS-3 were the *Haemophilus parasuis*-infected samples. **(C)** Comparisons of expression values of differentially expressed immunogenes between samples. The normalized MAS5 expression values were used here.

In particular, we found that the second principal component is a factor to classify the control and infected samples, of which the loading coefficients differed in their positive and negative signs (i.e., -0.205, -0.229 and −0.211 vs. 0.593, 0.094 and 0.066) (see the y-axis in Figure 1A). It means that the immunogenes loaded on the second principal component can service as covariate classifier or genomic signature that could distinguish the samples. In the first 20 genes with the largest positive loadings on the second axis (see Additional file 3: Table S2), except for PMM2, all members were also identified to be differentially expressed (see Figure 2B and 2C and section 2 of the Results and Discussion). This result indicates that the PCA analysis might provide an alternative or potential solution for identification of differentially expressed genes. To evaluate the reliability of the result, GeneSet Enrichment Analysis (GSEA) was further conducted. Figure 1D presents the enrichment plot for the gene set, and the detailed results of GSEA analysis are given in the Additional file 4. In this analysis, the Enrichment Score (ES), Normalized Enrichment Score (NES), Nominal p-value, False Discovery Rate (FDR) q-value and Family-Wise Error Rate (FWER) p-Value are estimated to be −0.938, -1.286, 0.00, 0.119 and 0.00, respectively. The result of the new PCA/GSEA combined method shows that a core set of 16 genes are increasingly expressed in the infected samples and could be used as an expression signature indicative of *H. parasuis* infection (see Additional file 4).

### Identification and GO annotation of differentially expressed immunogenes

One of the routine goals for transcriptomic analysis is to identify differences in the expression between phenotypic covariates of samples. To improve the detection power of the differential expression test [23,24], the inter-quartile range (IQR) filter was used to remove those uninformative genes. The differentially expressed immunogenes between the control and H. parasuis-infected groups were identified by empirical Bayes correction of the linear model [38], in which the cutoffs of p-value and logFC (log2-fold-change) were set as 0.05 and 1, respectively. The logFC, AveExpr (average log2-expression), t-statistic, p-value, adjusted p-value (q-value) and B-value (log odds value) for each gene can be found in the Additional file 5.

According to the cutoff criteria, a total of 36 immunogenes were detected to be differentially expressed. The estimates of t-value, p-value and logFC for all IQR-filtered immunogenes are given in Figure 2A. The hierarchical clustering presented in Figure 2B gives a distinct occurrence of differential expression pattern of these 36 immunogenes in which, compared to the control group, there were 9 down-regulated genes and 27 up-regulated genes (see Additional file 3: Table S2), respectively. The visual distinction between the up- and down-regulated immunogenes of the two-way hierarchical clustering pattern supports the reliability of the results of the differential expression test. In addition, a comparison of expression values of differentially expressed immunogenes between samples is displayed in Figure 2C, from which one can observe that the *H. parasuis* infection has mainly resulted in increased activation of immunogenes.

**Figure 3:**
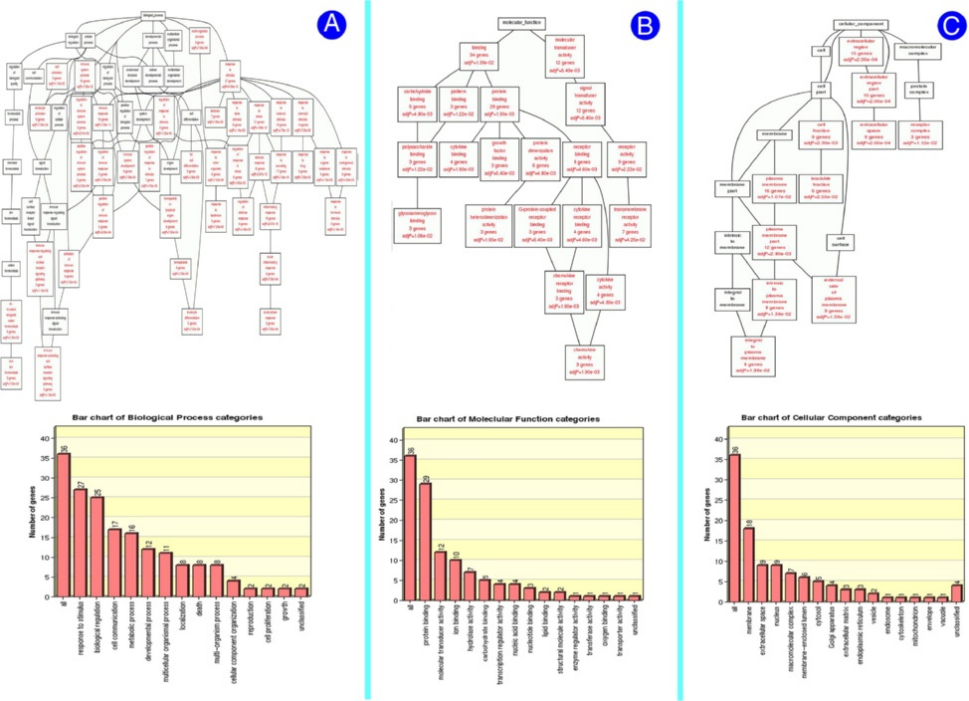
**GO annotations for differentially expressed immunogenes. In the hierarchically organized graphics, the target annotations are marked in red, which include GO BP/MF/CC names, numbers of genes and adjusted p values. (A)** Results of the Biological Process annotations. A total of 40 biological processes are involved. The lower histogram is a bar plot of the Biological Process annotations, including Biological Process names and the number of genes. **(B)** Results of the Molecular Function annotations of 21 molecular functions. The bar plot displays Molecular Function names and the number of genes. **(C)** Results of the Cellular Component annotations, in which 11 cellular components are displayed. The bar plot includes Cellular Component names and the number of genes.

Before Gene Ontology (GO) annotations, all of the immunogenes were converted into human gene symbols because of the relative scarcity of pig gene resources in the public databases. The annotation contents comprised the three major GO categories (Cellular Compartment or “CC”, Molecular Function or “MF”, and Biological Process or “BP”). As depicted in Figure 3, a graphical presentation of the GO annotations shows that all of the presented nomenclatures for BP, MF, and CC are graphically organized in systematic, hierarchical structures. If without considering the inclusive relationships of the upper hierarchical layers with the lower hierarchical ones, the 36 differentially expressed genes were associated with 40 biological processes. These processes mainly involve the immune processes, e.g., the immune system process, the leukocyte activation, the inflammatory response, the response to bacterium, and the immune system development. From the MF annotation results, there were 21 molecular functions identified. The majority of the 36 differentially expressed genes were linked to 2 classifications at the top hierarchical layer of nomenclatures of molecular functions, which include binding (34 genes involved) and molecular transducer activity (12 genes involved). The result of CC annotations revealed 11 cellular components, in which most of the immunogenes were preferentially assigned to the branch of plasma membrane.

### Reconstruction, visualization, and statistical evaluation of the immunogene network

Based on mutual information between immunogenes, the C3NET algorithm was used to estimate the adjacency matrix of the gene network [39]. Here, all of the immunogenes that passed the IQR filter were used to make the network inference. There are two reasons why we used the IQR-passed genes rather than the differentially expressed genes for network inference: 1) the number of differentially expressed immunogenes was relatively small, and, more importantly, the differential expression revealed the dimension-reduced or projected relationships between genes on the phenotypic covariate axis, which was not equivalent to the context of high-dimensional relationships of members in the network; and 2) it is accepted that not all members from a network will be simultaneously detected to be differentially expressed in real biological samples, and thus a network reconstructed only by differentially expressed genes was inadequate to depict the real topological architecture.

**Figure 4:**
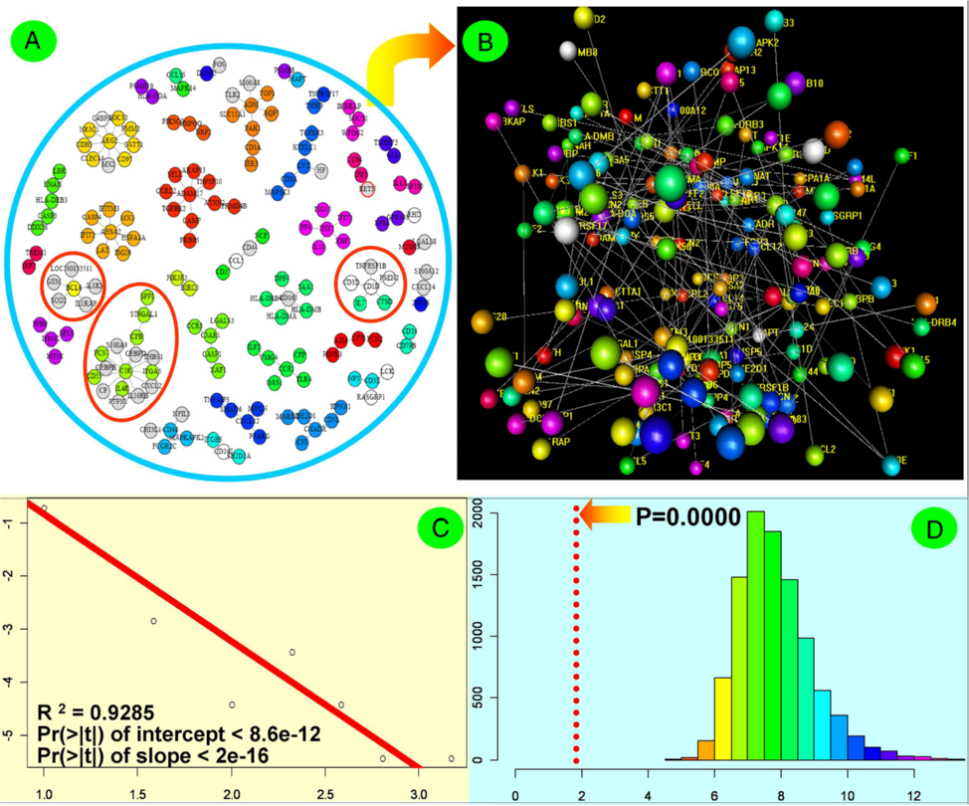
**Visualization and statistical evaluation of the compartmentalized network diagram. (A)** 2D plot for network topological structure. The white vertices are the downregulated, differentially expressed immunogenes, and the grey vertices are the upregulated genes. **(B)** 3D plot for network topological structure. In this sub-scenario, balls denote vertices (immunogenes), and lines denote edges (putative regulations) between two immunogenes. **(C)** Test of the scale-free property of the immunogene network. The logarithmic forms of degrees and their probabilities were linearly fitted. The red line represents the best fit. **(D)** Test of the average path length of immunogene network. The histogram is the distribution of the average path length of random graphs from 10000-time simulations. The vertical broken red line locates at x = 1.911, and it represents the estimation of the average path length of the reconstructed immunogene network.

Based on this modified routine, therefore, the planar and three-dimensional graphical representations of the network components are given in Figures 4A and 4B, and we can see that the network has incoherent topological characteristics where there are 35 total clusters (cliques) involved. The largest cluster, centered on C1R, is made up of 16 members and the clusters with their hub's degrees equal to or above 3 covers approximately 60% immunogenes. In addition to the known immunogenes involved in bacterial infections (e.g., S100A8 and IL4R) [40], most of them are the previously unreported members participating in the *H. parasuis*-induced network. Having obtained the topological structure of the inferred network, we further estimated the quantitative topological parameters. For a global quantitative evaluation of the reconstructed network, Table 1 gives the majority of topological parameters, which includes network density, diameter, node degree, average path length, hub score, betweenness, and closeness. Among these network cliques, the C1R-centered clique has the highest node degree, betweenness score and closeness. In general, some topological parameters of gene network can be endued with concrete biological meaning. For example, the node degree reveals the linking density (or regulatory intensity) between genes, and according to the principle of information theory, the betweenness score could be interpreted as the gene’s power to control the network. The closeness, one measure of network centralities, has the similar biological meaning of the betweenness score. Given the results of network parameters, the C1R-centered clique could be considered as the most important component of the *H. parasuis*-induced network.

**Table 1:** Topological parameters for the immunogene network

**Parameter**	**Estimate**	**Parameter**	**Estimate**
Density	0.009	Diameter	16.118
Betweenness score (edge)^*^	6.731	Degree^*^	1.567
Betweenness score (vertex)^*^	2.515	Vertex closeness^*^	0.006
Average path length	1.911	Burt’s constraint score^*^	0.863
Graph strength	8.368	Modularity value^*^	0.619
Dice similarity coefficient^*^	0.022	Kleinberg’s hub score	13.090

* denotes the mean of estimates.

The holistic properties of the network topology were further evaluated. The logarithmic forms of degrees and their probabilities (proportions) are graphically plotted in Figure 4C, two of which obviously represent a linear relationship. This means that the degree distribution satisfies a power law. If the degree distribution follows a power law, we say that the network is scale-free [41]. So, it can conclude that the network we reconstructed in this study is scale-free. Figure 4D displays the distribution of average path lengths of random networks (10000-time simulations based on the Erdös-Renyi model [42]), in which the vertical broken red line locates the estimation of average path length of the *H. parasuis* infection network. The location of the vertical broken red line indicates that the reconstructed network has a much smaller average path length than those of simulated random network. In addition, the clustering coefficient of the *H. parasuis* infection network is estimated to be zero, which is also much smaller than the average value of those from the randomly simulated networks. According to the complex network theory, the estimation results of average path length and clustering coefficient suggest that the immune network we reconstructed is not small-world.

There are two possibilities to explain these results. One is that the nodes in the reconstructed network did not cover the list of all potential immunogenes, and the absence of absent members decreases the global connectivity of the network. The other is that not all real biological networks are always small-world, and the network of *H. parasuis* infection might be strongly segmented or compartmentalized. Regarding the latter possibility, similar to *Mycobacterium bovis* bacillus Calmette-Guerin [43], the *H. parasuis* infection might initiate independent signalling cascades of various immune regulatory pathways that lead to a sparse-splitting immunogene network. Although many biological networks have been proven to belong to the small-world category, there have also been studies to support the second possibility. These include the long-range interaction networks in protein structure [44], the metabolic network of E. coli as defined by atomic mappings [45], the KSHV PPI network [46], the global network of Avian Influenza outbreaks [47], the sequence-based chemoinformatics threshold networks for drug target [48], and the network for phenolic secondary metabolism of T. cacao [49]. Therefore, the small-world property might be typical of networks, but might not be true for all real biological networks.

### Detection of the infection-induced network components

Here, by combining gene network ideas with differential expression, the network components involving differentially expressed immunogenes are considered to participate in, or at least be tightly associated with, the biological process of *H. parasuis* infection. Through mapping the members of differentially expressed immunogenes into the reconstructed network, we found that there were seventeen hub genes (of which the degree is defined here to be not lower than three), in which nine hubs (i.e., C1R, ADM, ARG2, BCL6, CD46, CD3E, CD163, CD1D, and LYZ) were involved in differentially expressed immunogenes. Although they were identified as being involved in the infection process of *H. parasuis*, most of the hub immunogenes themselves had no significant expression changes in the differential expression test. There were only two members (i.e., CD1D and CD163) that were identified to be differentially expressed. As can be seen in Figure 4A, when challenged with *H. parasuis*, CD1D was down-regulated, coupling with down-regulations of CD3D and PSEN2 and up-regulation of TNFRSF1B. Despite up-regulation of CD163, its linked neighbours were not detected to have significant changes in statistics. Furthermore, there were a total of 16 network clusters that had included the differentially expressed genes, and the network clusters mediated by C1R, CD1D and BCL6 were involved in at least four differentially expressed genes (see Figure 4A). In these clusters, 6 clusters involved down-regulated genes and 13 involved up-regulated ones. This means that there are three clusters being involved in both down-regulated and up-regulated genes.

Use of expression data for prioritization of network components is one of the extended applications of gene network analysis. The goals of network-based prioritization approaches often involve ranking the importance of genes or of network components. According to the statistics of graph theory, one of the prioritization approaches is to rank the betweenness scores (one type of measures of centrality) of vertices and edges [50]. Following this principle, we found that C1R had the largest betweenness score, and there were 12 vertices with the betweenness scores more than 10 (see Table 2). From the results, it was found that the importance of genes ranked by the betweenness scores correlated with, but did not exactly match, the ranks of vertex degrees. There is also other documented evidence consistent with our finding that the vertex degree was not always reliable for determining the importance of network components [51]. Moreover, in the first 19 genes with larger betweenness scores, there were only two members belonging to differentially expressed genes. That is, the ranking order of betweenness scores was almost completely uncorrelated with the results of differential expression test, and the differentially expressed tests only provide a low accuracy for the prioritization of genes.

**Table 2:** Rank of the betweenness scores of edges and vertices

**Edges**	**Betweenness score**	**Vertices**	**Betweenness score**
CP -- C1R	39	C1R	102
FCN2 -- CFH	28	ARG2	36
S100A8 -- ADM	18	ADAM17	34
CAMP -- ADAM17	16	CFB	26
CCRL2 -- ADAM17	16	ADM	25
CEBPB -- C1R	15	AHSA2	21
CEBPD -- C1R	15	ST6GAL1	14
CFB -- C1R	15	PAK1	12
CXCL2 -- C1R	15	BCL6	10
FCN1 -- C1R	15	CD163	10
IL10RB -- C1R	15	CD1D	10
IL4R -- C1R	15	IFIT1	10
ITGA5 -- C1R	15	CCR1	9
PTPN1 -- C1R	15	ATXN2	8
S100A9 -- C1R	15	CAMP	8
THBS1 -- C1R	15	CXADR	8
CASP1 -- C5AR1	15	LYZ	8
TNFRSF17 -- THBD	15	CD1A	7
CD3D -- CD1D	14	CD46	6

In the reconstructed network, there were 19 edges with betweenness scores greater than 10. According to the estimates of betweenness scores, the importance of the edge between CP and C1R was found to be much higher than others. Except for the edge between C1R and CD55 (only estimated as 1), the betweenness scores of all edges linked to C1R were found to be equal to or greater than 15. More importantly, the C1R-mediated network cluster was also involved in the largest number of differentially expressed immunogenes. It is known that C1R is one of the early complement proteases, which plays a crucial role in immune responses against microbial pathogens. Based on the network-based prioritization, despite not being differentially expressed gene, C1R and its co-expressed genes are considered to be the most important network components associated with *H. parasuis* challenge. In our opinion, both C1R and its co-expressed members might play key roles in the coordination of host defense mechanisms against the *H. parasuis* infection.

### Evaluation of immunogenes with the higher betweenness scores (at least 10) and bioinformatic validation of the C1R-centered clique

**Figure 5:**
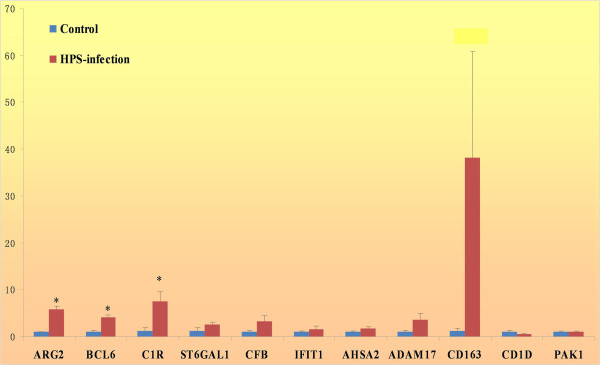
**qPCR evaluation of the twelve immunogenes with betweenness scores greater than 10 in the control and HPS-infected samples.** ARG2, BCL6, C1R, ST6GAL1, CFB, IFIT1, ASHA2, ADAM17, ADM and CD163 were up-regulated in HPS-infected samples (red bars) compared with normal controls (blue bars), and CD1D and PAK1 were down-regulated. The vertical axis denotes the fold change calculated based on the mean intensity value from each individual within each group using the comparative Ct method. The expression differences were analyzed with T-tests. Asterisks (*) above the bars represent that the expression differences of the genes between the control and infected groups are significant at the level of *p* ≤ 0.05.

To confirm the *in vivo* expressions of the immunogenes with the betweenness score equal to or more than 10, a total of 12 genes were validated by quantitative real-time PCR (qPCR). The primer sequence, melting temperature and product sizes for 12 immunogenes in the qPCR analysis are shown in Table 3. The detailed results for each gene are listed in the Additional file 6. The results for a panel of these 12 genes show that, with the exception of the down-regulated CD1D and PAK2, most of them were up-regulated in infected samples (see Figure 5), which is in agreement with the microarray analytical results.

**Table 3:** Primer sequences, melting temperatures and product sizes for twelve immunogenes in the qPCR analysis

**Gene symbol**	**Primer sequence (5'-3')**	**Target size (bp)**	**Tm (°C)**
ADAM17	Sense: TGGCAGACAACATCGTGGG	106	59
	Anti-sense: GGGCTTGATGATGCGAACG		
ARG2	Sense: GCATTCCGCCCCGAAG	233	65
	Anti-sense:CCACTGAGCGAGGATTCACT		
C1R	Sense: TGTGGACCTGGATGAATGT	101	59
	Anti-sense: AATAGCCGCCGATGTAGTT		
CFB	Sense: CATTCATGATATTCGGGACCT	92	59
	Anti-sense: GCCCAACCCCAAACACGTA		
CD1D	Sense: GTGGCTCCTCTACGACATCT	88	55
	Anti-sense: TTCAGGCTTCACTTGCTTCTC		
BCL6	Sense: TTGTCATAGTGGTGAGCCGAGAG	168	65
	Anti-sense: TGAAGTCCAGGAGGATGCAGAAC		
ADM	Sense: CGCTACCGCCAGAGTATGAACA	121	59
	Anti-sense: CGTCCTTGTCTTTGTCCGTGAAC		
IFIT1	Sense: AATTAGCCACAGGTCATT	144	55
	Anti-sense: ATTCCATACACAACACTCT		
ASHA2	Sense: AATCTCCTGCTATATTAGAAG	126	55
	Anti-sense: AGTTCCTACATCTCCATT		
PAK1	Sense: CACTTCCTATTACTCCAACT	113	59
	Anti-sense: CTTCTTCTTCTGCTTCTCA		
ST6GAL1	Sense: GACATCATTCAAGAAATCTC	164	59
	Anti-sense: AAGAACTTCTGGTAGTAGTA		
RPL32 ^a^	Sense: CGGAAGTTTCTGGTACACAATGTAA	94	55-65
	Anti-sense: TGGAAGAGACGTTGTGAGCAA		

^a^ The transcript levels of RPL13 were used for sample normalization.

Due to the mathematical essence of a static network based on mutual information, a network clique simply defines a functionally related gene set, which means that a clique is essentially denotes the extent of functional coupling expressions of a gene set. Given this, we focused on the most important clique and conducted a pathway enrichment analysis (PEA) to detect the possible existence of direct or indirect regulation among the members appearing in the C1R-centered clique. Here, the web tool Gene Set Analysis Toolkit V2 was used to perform pathways enrichment analysis [52,53], and the parameters of enrichment analyses were set as follows: the statistical test used the hypergeometric method, the multiple test adjustment used the Benjamini and Hochberg method, the significance level was set to 0.05, and the minimum number of genes for a category was set to 2. In terms of option selection, the pathway database resources included the Pathway Commons, Wikipathways, and KEGG pathway databases.

The results of pathway enrichment analysis are listed in Table 4, which identifies genes in the C1R-centered clique that were significantly enriched in more than 30 signaling pathways. The existence of coupling relationships between these immunogenes is primarily supported by the bioinformatic analysis, and there are surely potential direct or indirect regulations between the members of the C1R-centered clique. In particular, by combining Tables 2 and 4, the explicit graphical connection of “CFB -- C1R” was found to be enriched in several known pathways, which include the initial triggering of complement (DB_ID:505), the complement cascade (DB_ID:503), and the innate immunity signaling (DB_ID:804) in Pathway Commons database, and the complement and coagulation cascades (04610) in the KEGG pathway database. The PEA analysis primarily provides a digital validation of the reliability of the C1R-centered clique.

**Table 4:** Bioinformatic analysis to identify the existence of potential regulations in the C1R-mediated cluster through pathway enrichment analysis

**Pathway names**	**Enriched genes in pathway**	**P value**	**Adjust P value**
**1. Pathway Commons database**			
Inflammatory Response Pathway	IL4R, THBS1	4.42e-05	0.0001
Focal Adhesion	ITGA5, SPP1, THBS1	2.93e-05	0.0001
TGF Beta Signaling Pathway	SPP1, THBS1	0.0001	0.0002
Senescence and Autophagy	CEBPB, THBS1	0.0002	0.0003
Adipogenesis	CEBPB, CEBPD	0.0009	0.0009
Initial triggering of complement	C1R, CFB	6.60e-05	0.0006
Complement cascade	C1R, CFB	6.60e-05	0.0006
Syndecan-4-mediated signaling events	ITGA5, THBS1	0.0003	0.0019
IL6-mediated signaling events	CEBPB, CEBPD	0.0003	0.0019
Exocytosis of Alpha granule	ITGA5, THBS1	0.0003	0.0019
Platelet degranulation	ITGA5, THBS1	0.0004	0.0024
IL4-mediated signaling events	CEBPB, IL4R	0.0005	0.0026
Response to elevated platelet cytosolic Ca^++^	ITGA5, THBS1	0.0005	0.0026
Innate Immunity Signaling	C1R, CFB	0.0007	0.0030
Platelet Activation	ITGA5, THBS1	0.0008	0.0033
Regulation of p38-alpha and p38-beta	CEBPB, PTPN1	0.0028	0.0104
Proteogylcan syndecan-mediated signaling events	ITGA5, THBS1	0.0035	0.0124
p38 MAPK signaling pathway	CEBPB, PTPN1	0.0037	0.0125
Plasma membrane estrogen receptor signaling	CEBPB, PTPN1	0.0048	0.0150
BMP receptor signaling	CEBPB, PTPN1	0.0053	0.0159
Regulation of nuclear SMAD2/3 signaling	CEBPB, PTPN1	0.0101	0.0246
TGF-beta receptor signaling	CEBPB, PTPN1	0.0101	0.0246
Regulation of cytoplasmic and nuclear SMAD2/3 signaling	CEBPB, PTPN1	0.0101	0.0246
Glypican 1 network	CEBPB, PTPN1	0.0224	0.0380
Glypican pathway	CEBPB, PTPN1	0.0258	0.0386
**2. Wikipathways database**			
Inflammatory Response Pathway	IL4R, THBS1	4.42e-05	0.0001
Focal Adhesion	ITGA5, SPP1, THBS1	2.93e-05	0.0001
TGF Beta Signaling Pathway	SPP1, THBS1	0.0001	0.0002
Senescence and Autophagy	CEBPB, THBS1	0.0002	0.0003
Adipogenesis	CEBPB, CEBPD	0.0009	0.0009
**3. KEGG database**			
ECM-receptor interaction	ITGA5, SPP1, THBS1	2.75e-06	1.65e-05
Focal adhesion	ITGA5, SPP1, THBS1	3.76e-05	0.0001
Cytokine-cytokine receptor interaction	IL4R, CXCL2, IL10RB	8.72e-05	0.0002
Complement and coagulation cascades	C1R, CFB	0.0002	0.0003
Hematopoietic cell lineage	ITGA5, IL4R	0.0004	0.0005
Jak-STAT signaling pathway	IL4R, IL10RB	0.0012	0.0012

## Conclusions

Recently, many studies have focused on the *H. parasuis*, a model Gram-negative bacterium. However, among these studies, none have paid attention to the host immune network and its quantitative topology. In this work, by targeting the spleen immunogenome, we have reconstructed the immune network and probed the network topology parameters that characterize the immunogenome-wide expression behaviors in response to *H. parasuis* infection. Our analyses suggest that the reconstructed immune network is scale-free but not small-world. To our knowledge, we report the first investigation into the immunogenome-focused network biology analysis of *H. parasuis* infection. Compared with our previous investigation [10], the immunogenome-focused study has mined much new information about the host infection biology of Gram-negative bacterium *H. parasuis*. Although the number of replicates only met the basic requirements for sample size of microarray studies, the results showed that the immunogenome-focused strategy we used has worked efficiently. In addition, our results are valuable and may have potential applications, for instance, our results might provide new or potential targets for interrupting or alleviating the course of bacterial infections.

In summary, we used network biology approaches to quantitatively characterize the nature of immune network responding to *H. parasuis* infection. Our results for the first time revealed an immunogenome-focused network of porcine spleen challenged with *H. parasuis*, which also provide a step toward a network biology-based understanding of infection with the Gram-negative *bacilli* in mammals.

## Methods

### Data source

The basic raw data of six Affymetrix chips used for the extraction of immunogenome data came from our previous study [10], in which the spleen tissues of three HPS infected piglets and three controls were individually used for the experiment. The Affymetrix chip data has been deposited in the NCBI Gene Expression Omnibus (GEO) database under the GSE series accession number GSE11787.

### Web resources and tools

The web resources and tools used in this study mainly include the Affymetrix technical files (http://www.affymetrix.com/support/index.affx); the R packages (http://www.r-project.org/); the Bioconductor packages (http://www.bioconductor.org); the annotation tools of WebGestalt (http://bioinfo.vanderbilt.edu/webgestalt/); the KEGG database (http://www.genome.jp/kegg/); the reactome database (http://www.reactome.org/); the GSEA analysis tool (http://www.broadinstitute.org/gsea/downloads.jsp); the igraph R package (http://cneurocvs.rmki.kfki.hu/igraph/); and two-way hierarchical clustering (http://faculty.ucr.edu/~tgirke/Documents/R_BioCond/My_R_Scripts/my.colorFct.R).

### Statistical analyses

R/Bioconductor is open-source, freely available, and widely used for high-throughput data analyses in a variety of biological fields. In this study, the R/Bioconductor packages and self-written procedures in R statistical environment (available upon request: zhumengjin@mail.hzau.edu.cn) were used to perform the statistical analyses. The affy package was used to perform the low-level processing processes that included quality control, background correction, PM correction, summarization, normalization and probeset filtering. The non-specific filtering, principal components analysis and differentially expressed test were mainly realized by the packages of genefilter, FactoMineR, and limma. The javaGSEA Desktop Application tool was used for GSEA analysis. The packages used for graphical representations mainly included graphics, stats, MASS, misc3d, plotrix and RColorBrewer. The packages of c3net and igraph, and self-written procedures, were used for network reconstruction, including estimations of mutual information, adjacency matrix, network parameters and network topological properties, and graphical representations. All of these packages are free and can be downloaded from the websites: http://www.bioconductor.org and http://cran.r-project.org.

### Quantitative real-time PCR

Two-step quantitative RT-PCR (qPCR) was performed on the same spleen RNA samples used for the microarray experiments. Total RNA were treated with DNaseI (Tubo kit, Ambion) and reverse transcribed using the RevertAid™ First Strand cDNA Synthesis Kit (Fermentas) according to the manufacturer's instructions. We used the ribosomal protein L32 (RPL32) gene as an internal control. qPCR was run on the LightCycler® 480 Real-Time PCR System (Roche), in which the SYBR® Green Real-time PCR Master Mix (TOYOBO CO., LTD, Japan) was used as the readout. The cycling parameters were as follows: 95x°C, 2 min; 95°C, 15 s; X°C as appropriate, 15 s, where X is 55, 59 or 65°C depending on the primer pair used; and 72°C, 20 s for 45 cycles. After PCR, a melt-curve analysis of each primer pair was carried out to verify the specificity of the PCR assay. The correct fragment sizes of the PCR products were confirmed using agarose gel electrophoresis (2%). Each primer set amplified a single product as indicated by a single peak present for each gene during melting curve analyses. The relative quantitative gene expression level was evaluated using the comparative Ct method. The ΔCt values were calculated by subtracting the RPL32 Ct value for each sample from the target Ct value of that sample. The duplicates for each sample were averaged, and pairwise t tests were conducted to determine differential expression between control and infection. In all qPCR analyses, the significance level was set at *p* ≤ 0.05.

## Abbreviations

HPS: Haemophilus parasuis; H. parasuis: Haemophilus parasuis; PCA: Principal components analysis; IQR: Inter-quartile range; GSEA: Geneset enrichment analysis; ES: Enrichment score; NES: Normalized enrichment score; FDR: False discovery rate; FWER: Family-wise error rate; logFC: Log fold change; KEGG: Kyoto Encyclopedia of Genes and Genomes; GO: Gene ontology; CC: Cellular compartment; MF: Molecular function; BP: Biological process; 2D: Two-dimensional; 3D: Three-dimensional; PEA: Pathway Enrichment Analysis; PPI: Protein-protein interaction; qPCR: Quantitative real-time polymerase chain reaction.

## Competing interests

The authors declare that they have no competing interests.

## Authors’ contributions

MJZ and SHZ designed experiments; MZ and XDL collected and annotated the immunogenes data; MJZ and MZ analyzed data; MZ, XDL, XYL and HJ performed the experiments; RZ and HBC contributed to the Affymetrix chip experiment; MJZ wrote the original manuscript; SHZ, XYL, MZ, XDL and RZ reviewed and modified the paper. All authors have read and approved the manuscript.

## Supplementary Material

Additional file 1 **The list of immune pathways used for extraction of immunogenes.** The list includes the immune pathway names, the database sources, and the numbers of immunogene for each pathway. There are totally 62 immune pathways involved. Click here for file

Additional file 2 **The corresponding list of Affymetrix probesets and 1,999 transcripts of immunogenes.** By using the raw data provided by Dr. Hu, this file includes Affy ID, ITC ID, RefSeq ID, RefSeq Desc, Gene ID, Gene Sym, Tax, A2I Score, A2I Evaule, I2R Score, and I2R Evalue. Click here for file

Additional file 3: Table S1 Descriptive statistical parameters for expression values of immunogenes. The descriptive statistical parameters in Table S1 include mean, minimum, maximum, variance, coefficient of variation, and mean absolute deviation. Click here for file

Additional file 4 **The GSEA analysis results of gene set of the first 20 genes with the largest positive loadings on the second principal component.** This file shows Affy ID, description, gene symbol, rank in gene list, rank metric score, running ES, and core enrichment. Click here for file

Additional file 5 The logFC, AveExpr, t-statistic, p-value, adjusted p-value (q-value) and B-value for each gene. Click here for file

Additional file 6 **The raw data and statistical results for the results of qPCR analysis.** Additional file 6 includes gene name, CT of infection and its average, internal control of infection and its average, ΔCT(inf), CT of control and its average, internal control of control and its average, ΔCT(ctl) and its average, ΔΔCT of inf and its statistical parameters, ΔΔCT of ctl and its statistical parameters, and p-value of *t*-test. Click here for file
