# Supplementary material for: Systems infection biology: a compartmentalized immune network of pig spleen challenged with Haemophilus parasuis
Source: BMC Genomics. 2013 Jan 22;14:46. doi: 10.1186/1471-2164-14-46 (PMC3610166; doi:10.1186/1471-2164-14-46)
Supplement: Additional file 3: Table S1 — Descriptive statistical parameters for expression values of immunogenes. The descriptive statistical parameters in Table S1 include mean, minimum, maximum, variance, coefficient of variation, and mean absolute deviation. [file 1471-2164-14-46-S3.doc]

Table S1. Descriptive statistical parameters for expression values of immunogenes

| Parameters | Ctr-1 | Ctr-2 | Ctr-3 | HPS-1 | HPS-2 | HPS-3 |
| --- | --- | --- | --- | --- | --- | --- |
| Mean | 6.716 | 6.709 | 6.756 | 6.652 | 6.825 | 6.807 |
| Min | 2.575 | 2.789 | 2.693 | 2.657 | 2.632 | 2.739 |
| Max | 14.155 | 14.007 | 14.214 | 14.014 | 14.460 | 14.301 |
| Var | 6.044 | 5.860 | 6.068 | 5.733 | 6.319 | 6.374 |
| CV | 0.366 | 0.361 | 0.365 | 0.360 | 0.368 | 0.371 |
| Mad | 2.985 | 2.952 | 2.981 | 2.828 | 3.093 | 3.148 |

Note: Ctr = control group, HPS = Haemophilus parasuis-infected group. Numbers following the short dash symbol mean technical replicates. Min = minimum, Max = maximum, Var = variance, CV = coefficient of variation, and Mad = mean absolute deviation.

Table S2. Immunogene lists of the second principal component and differentially expressed genes

| 20 genes with the largest positive loadings | down-regulated genes | up-regulated genes |
| --- | --- | --- |
| S100A9, RETN, CXCL2, S100A8, S100A12, SOD2, IL1RAP, CP, CD163, LOC100133511, TNFRSF1B, CEBPB, PMM2, CEBPD, GEM, TLR2, MX2, IL10RB, CHI3L1, and IL1RN | C4BPA, RHD, CD1D, CD3D, RASGRP1, CCL5, CD247, PSEN2, and LCK | PTPN1, FOS, THBS1, CXCL14, TLR2, TNFRSF1B, CD44, LGALS8, MX2, IL10RB, IL1RN, CEBPB, NFIL3, LOC100133511, CEBPD, GEM, HP, CD163, SOD2, CHI3L1, CP, IL1RAP, CXCL2, S100A12, S100A8, RETN, and S100A9 |
|
|
|
|
|

Note: Ctr = control group, HPS = Haemophilus parasuis-infected group. Numbers following the short dash symbol mean technical replicates. Min = minimum, Max = maximum, Var = variance, CV = coefficient of variation, and Mad = mean absolute deviation.
